# Miniaturized CO_2_ Gas Sensor Using 20% ScAlN-Based Pyroelectric Detector

**DOI:** 10.1021/acssensors.2c00980

**Published:** 2022-08-09

**Authors:** Doris Keh Ting Ng, Linfang Xu, Weiguo Chen, Huanhuan Wang, Zhonghua Gu, Xavier Xujie Chia, Yuan Hsing Fu, Norhanani Jaafar, Chong Pei Ho, Tantan Zhang, Qingxin Zhang, Lennon Yao Ting Lee

**Affiliations:** † Institute of Microelectronics, A*STAR (Agency for Science, Technology and Research), 2 Fusionopolis Way, #08-02, Innovis Tower, Singapore 138634, Singapore; ‡ Photonics Devices and Systems Group, Engineering Product Development, Singapore University of Technology and Design, 8 Somapah Road, Singapore 487372, Singapore

**Keywords:** pyroelectric detector, scandium aluminum nitride (ScAlN), abnormally oriented grains, CO_2_ gas sensor, MEMS, CMOS compatible, nondispersive infrared

## Abstract

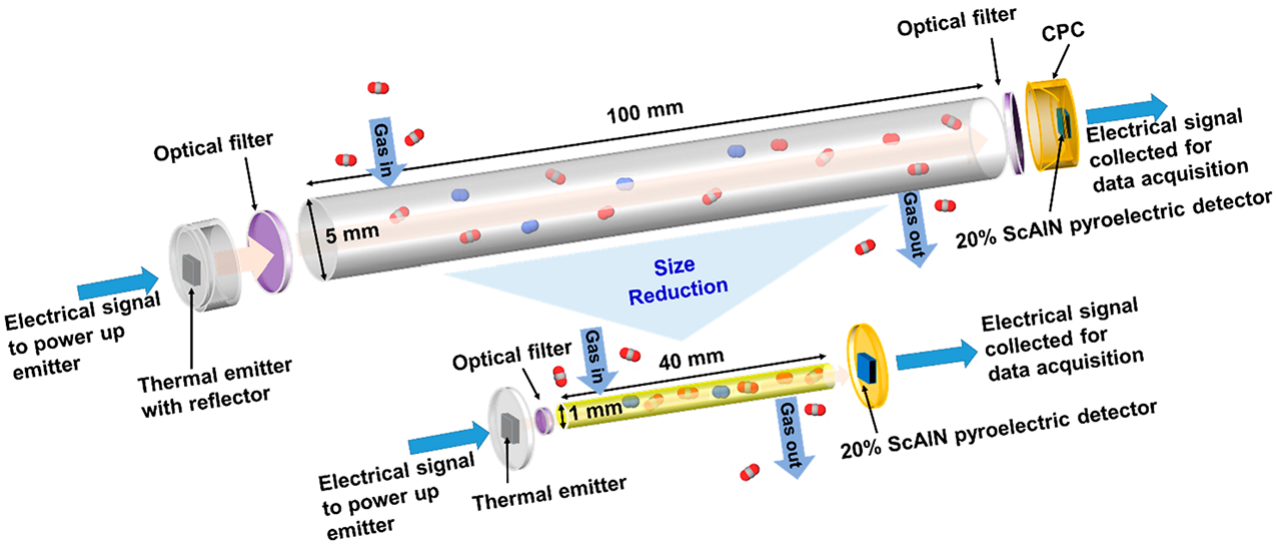

NDIR CO_2_ gas sensors using a 10-cm-long gas channel and CMOS-compatible 12% doped ScAlN pyroelectric detector have previously demonstrated detection limits down to 25 ppm and fast response time of ∼2 s. Here, we increase the doping concentration of Sc to 20% in our ScAlN-based pyroelectric detector and miniaturize the gas channel by ∼65× volume with length reduction from 10 to 4 cm and diameter reduction from 5 to 1 mm. The CMOS-compatible 20% ScAlN-based pyroelectric detectors are fabricated over 8-in. wafers, allowing cost reduction leveraging on semiconductor manufacturing. Cross-sectional TEM images show the presence of abnormally oriented grains in the 20% ScAlN sensing layer in the pyroelectric detector stack. Optically, the absorption spectrum of the pyroelectric detector stack across the mid-infrared wavelength region shows ∼50% absorption at the CO_2_ absorption wavelength of 4.26 μm. The pyroelectric coefficient of these 20% ScAlN with abnormally oriented grains shows, in general, a higher value compared to that for 12% ScAlN. While keeping the temperature variation constant at 2 °C, we note that the pyroelectric coefficient seems to increase with background temperature. CO_2_ gas responses are measured for 20% ScAlN-based pyroelectric detectors in both 10-cm-long and 4-cm-long gas channels, respectively. The results show that for the miniaturized CO_2_ gas sensor, we are able to measure the gas response from 5000 ppm down to 100 ppm of CO_2_ gas concentration with CO_2_ gas response time of ∼5 s, sufficient for practical applications as the average outdoor CO_2_ level is ∼400 ppm. The selectivity of this miniaturized CO_2_ gas sensor is also tested by mixing CO_2_ with nitrogen and 49% sulfur hexafluoride, respectively. The results show high selectivity to CO_2_ with nitrogen and 49% sulfur hexafluoride each causing a minimum ∼0.39% and ∼0.36% signal voltage change, respectively. These results bring promise to compact and miniature low cost CO_2_ gas sensors based on pyroelectric detectors, which could possibly be integrated with consumer electronics for real-time air quality monitoring.

Carbon dioxide (CO_2_) sensors have been of interest in recent years to monitor air quality in enclosed crowded spaces. The need for well ventilated spaces is especially important these days to ensure good air quality, so as to reduce the transmission of SARS-CoV-2/COVID-19 (severe acute respiratory syndrome coronavirus-2/coronavirus disease 2019). Even before the COVID-19 pandemic, there were reports on the detrimental effects to human health with extended exposure to high concentrations of CO_2_.^1^ Specifically, elevated levels of CO_2_ (>1000 ppm of CO_2_ gas concentration) in enclosed spaces will result in sick building syndrome (SBS),^2^ with occupants experiencing drowsiness, headaches, and difficulties in concentrating. With the presence of CO_2_ sensors, one would be able to monitor in real-time the quality of air, raising awareness on the quality of the air we breathe in. This would become more of a reality if the CO_2_ gas sensors are low cost and miniature. Cost is an important factor, as CO_2_ gas sensors which are low cost can enable more sensors to be deployed for more accurate real-time CO_2_ level monitoring within a certain area. If these sensors are high cost, the number of sensors that can be deployed for air quality monitoring will be limited by the sensor price. Other than the cost of manufacturing the sensor, the operational cost such as the power required to operate the sensor and the temperature at which the sensor operates could also add to the cost. At the same time, functional flexibility of these CO_2_ sensors should also be considered, whether they can be modified with minimum cost or involve change to sense different gases. In general, there are many factors when considering the cost of a CO_2_ sensor. In addition to reducing its cost, the size of each sensor is also of importance, as a bulky sensor will make mass deployment difficult, not to mention that material cost might increase for a sensor with a bigger form factor. Targeting low-cost and miniature sensors will allow them to be easily integrated onto consumer electronics, allowing one to monitor CO_2_ levels in real-time.

Much effort has been put into the development of CO_2_ gas sensors for environmental monitoring. Types of CO_2_ sensors that have been reported include mid-infrared (mid-IR) sensors based on the nondispersive infrared (NDIR)^3−6^ technique, attenuated total reflection^7^ technique, chemiresistive^8^ sensors, amperometric^9^ sensors, acoustic^10^ sensors, and the adoption of metal–organic framework^11−13^ for improved gas sensing performance.

NDIR CO_2_ sensors are promising as they have been proven to have high selectivity and high stability^14−16^ with fast response time.^17,18^ However, they have also been reported to have a large footprint^13^ and are costly.^5^ We try to overcome these limitations by reducing the size of the gas channel of an NDIR CO_2_ sensor and using a broadband thermal emitter and scandium aluminum nitride (ScAlN)-based pyroelectric detector.

Pyroelectric detectors are thermal sensors that will produce an electrical signal when they experience an instantaneous temperature change. However, as pyroelectric materials are also piezoelectric, pyroelectric detectors will be affected by microphonic effects.^19,20^ Any mechanical stress encounter by the pyroelectric detector due to vibration or pressure will also produce an electrical signal output. In pyroelectric detectors, microphonic effects are undesirable, and it is important to minimize them. If the signal due to microphonic effects is too high, it can exceed the device signal for minimum thermal detection and the detection limit of the pyroelectric detector will be affected. To reduce microphonic effects, efforts have been made to reduce mechanical vibrations in the pyroelectric detectors. InfraTec GmbH, one of the major companies that develop and manufacture pyroelectric detectors, recommends using vibration dampers such as rubber connectors or elastic cables to minimize mechanical vibrations and to choose suitable electrical bandpass to reduce interferences from higher frequencies.^21^ Norkus et al.^20^ consider the device layout, mounting elements and introduce thermal isolation trenches, while Xu et al.^22^ use 3D-printing of inverted pyramid suspending architecture for the pyroelectric detectors to minimize the microphonic effect.

Studies leveraging pyroelectric effects include self-powered temperature sensors,^23^ solar energy harvesters,^24^ and driving wireless sensors.^25^ Here, we use a ScAlN-based pyroelectric detector for CO_2_ gas sensing. The thermal emitter and pyroelectric detector emits and receives light, respectively, over a wide wavelength range, hence allowing the flexibility to detect different types of gases by requiring only the change in optical filter to match the characteristic absorption wavelength of the gas targeted for detection. By using a broadband thermal emitter and a pyroelectric detector, we try to reduce the cost of our sensor by allowing it to have the flexibility to be used to detect different gases with only the change of an optical filter, rather than the source or the detector. The thermal emitter and pyroelectric detector operate at room temperature; hence no extra cost is required to create the stipulated temperature conditions. In addition, our ScAlN-based pyroelectric detectors are complementary metal oxide semiconductor (CMOS)-compatible.^26^ The ScAlN layer is deposited at a low thermal budget of ∼200 °C, and the detector devices are fabricated using semiconductor wafer level technology on 8-in. silicon (Si) wafers. This will allow for mass manufacturing and miniaturization, with many detectors fabricated on one 8-in. Si wafer, reducing the cost per detector, and allowing for monolithic integration with CMOS electronics. If we consider a detector with die size of 3 mm × 2 mm fabricated over an 8-in. wafer area and 80% yield, we estimate ∼4000 detector devices obtained from one wafer.

In this paper, we build a low cost, miniature NDIR CO_2_ gas sensor using a 20% Sc-doped AlN-based pyroelectric detector fabricated in-house with a 40-mm-long, 1-mm-diameter gas channel and a broadband thermal emitter (Axetris, EMIRS50 AT06 V). This gas sensor is reduced by ∼65 times in volume compared to its larger version 100-mm-long, 5-mm-diameter gas channel. We characterize the 20% doped ScAlN-based pyroelectric detector. Top view scanning electron microscopy (SEM) shows grainy detector surfaces, and cross-sectional transmission electron microscopy (TEM) images reveal abnormally oriented grains (AOGs) in the ScAlN sensing layer. This detector is further characterized optically and electrically. The absorption of the pyroelectric detector stack shows ∼50% absorption at the CO_2_ absorption wavelength of 4.26 μm. The pyroelectric coefficient is calculated to be ∼11.4 μC/m^2^ K when a temperature fluctuation of 2 °C (from 24 to 26 °C) is applied within ∼8 s to the pyroelectric detector using a Peltier heater. The pyroelectric coefficient obtained is higher than that of 12% ScAlN previously reported.^27^ We also note that as the set background temperature increases, the output current measured from the pyroelectric detector shows an increasing trend. This eventually corresponds to an increase in the calculated pyroelectric coefficient when measured at higher background temperature, ranging from ∼11.4 to ∼18.7 μC/m^2^ K. The CO_2_ gas sensing response is measured for this miniature CO_2_ gas sensor together with its larger version. For this miniature CO_2_ gas sensor, the results show a lower detection limit ∼100 ppm of CO_2_ gas concentration and response time ∼5 s. With the size of this current miniature gas sensor taken into account and compared with NDIR CO_2_ gas sensors reported in recent years, the performance of our miniature gas sensor using 20% ScAlN-based pyroelectric detector is comparable, if not better. The selectivity of this miniature CO_2_ gas sensor is also tested by mixing CO_2_ with nitrogen (N_2_) and 49% sulfur hexafluoride (SF_6_), respectively, instead of synthetic air. The results show high selectivity to CO_2_ with N_2_ and SF_6_ causing a minimum signal voltage change of ∼0.39% and ∼0.36%, respectively. Finally, we do a comparison of the performance of our miniaturized CO_2_ gas sensor with off-the-shelf CO_2_ gas sensors in terms of detection range, response time, and interference from other gases. Overall, the gas sensing performance of the miniature CO_2_ gas sensor obtained is encouraging and signifies the possibility of miniaturizing the NDIR CO_2_ gas sensor, which could in time be integrated with consumer electronics.

## Experimental Section

### 20% ScAlN-Based Pyroelectric Detector

The fabrication of this 20% ScAlN-based pyroelectric detector follows similar steps^27−29^ as what had previously been described for realization of 12% ScAlN-based pyroelectric detectors. The top electrode is titanium nitride (TiN), while the bottom electrode is molybdenum (Mo). The ScAlN sensing layer is sandwiched between the top and bottom electrode. Here, the sensing layer is 20% Sc-doped AlN, and this layer is deposited using the physical vapor deposition process at a deposition temperature of ∼200 °C. Above the top TiN electrode is a dielectric absorber stack. Aluminum (Al) is used for the metal contact layers for the top and bottom electrodes. Beneath the bottom Mo electrode is a layer of silicon dioxide (SiO_2_) patterned with waffle-like structures^27,30^ to help increase mechanical stiffness of the pyroelectric detector membrane stack and act as a damper to reduce microphonic effects.

### Electrical Measurement of 20% ScAlN-Based Pyroelectric Detector

The 20% ScAlN-based pyroelectric detector with AOGs in the ScAlN sensing layer is placed in a bathtub hybrid package housing wire-bonded to 2 different leads. This bathtub hybrid package is then placed on a Peltier heater using thermal grease. This heater is connected to a benchtop temperature controller. The detector in the bathtub hybrid package is connected to a current amplifier with gain set at 10^9^ V/A. The current amplifier is connected to an oscilloscope to capture the waveform generated when the temperature change is triggered. The temperature controller is then set to the respective background temperature. During measurement, the temperature controller is set to trigger a 2 °C temperature change to the Peltier heater. The Peltier then heats by 2 °C within ∼8 s. The pyroelectric detector detects the temperature fluctuation and generates a pyroelectric current waveform captured by the oscilloscope.

### Gas Sensor Assembly

**Figure 1:**
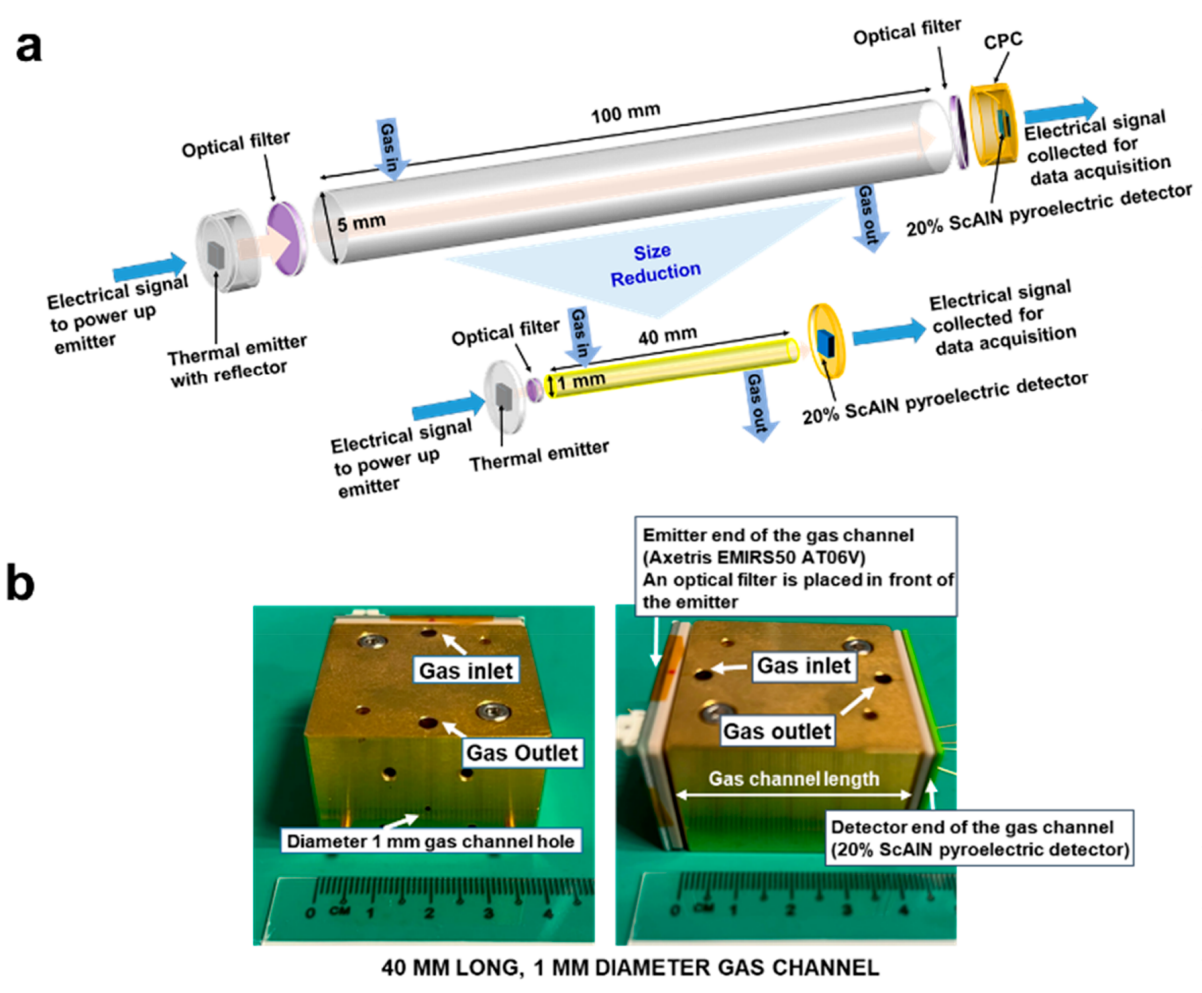
(a) Schematics showing the configuration of NDIR CO_2_ sensors used (partially exploded view drawing). A thermal emitter is used as the source, and 20% ScAlN pyroelectric detector with AOGs is used as the detector. To miniaturize the system, the gas channel is reduced from 100 mm length to 40 mm length, with diameter from 5 mm reduced to 1 mm, respectively. Optical filters are inserted along the light path to allow light of wavelength ∼4.26 μm to pass through, as 4.26 μm is the molecular characteristic wavelength that CO_2_ absorbs. (b) Photo images showing the reduced size gas channel with the left image revealing the gas channel hole with diameter 1 mm where the IR light will travel through and the right image presenting the assembly of the gas sensor. The effective region of the gas channel is the 1-mm-diameter gas channel hole and the 40-mm-long gas channel length. The thermal emitter source and ∼4.26 μm wavelength optical filter are on one end of the gas channel, and the 20% ScAlN-based pyroelectric detector is on the other end of the gas channel. The emitter and detector are positioned with the center of the heater area and sensing area right in front of the 1-mm-diameter gas channel, held together by micromachined fixtures and screws. There are 2 holes on top of the gas channel for the gas inlet and outlet.

Figure 1a shows schematics of two CO_2_ gas sensors. We use an NDIR gas sensing technique which usually consists of a source, optical filter, gas channel, and detector system. A broadband thermal emitter (Axetris, EMIRS50 AT06 V) with heater area ∼0.64 mm^2^, set to modulate at 17.4 Hz, is used in our case to emit light across wavelengths from 2 to 14 μm. This is to reduce cost where one emitter source could be used to detect any gases that have characteristic absorption wavelength falling in the range 2–14 μm, hence increasing the functional flexibility of this sensor. We only need to change the narrow bandpass optical filter that will allow specific wavelength to pass through, which would correspond to the absorption wavelength of the gas to be detected. In our case, as we are detecting CO_2_, we choose an optical filter of center wavelength ∼4.26 μm with half power bandwidth ∼180 nm. On the detector end is a 20% ScAlN-based pyroelectric detector with AOGs. The sensing area of the detector is ∼0.29 mm^2^. A compound parabolic collector (CPC) could be used to concentrate the optical signal collected onto the detector. Between the source and the detector is a metal gas channel. Initially the gas channel is of diameter ∼5 mm, length ∼100 mm, and volume ∼1.96 cm^3^. To reduce the gas sensor size, we further shrink the gas channel from diameter ∼5 mm down to ∼1 mm with channel length shortened from ∼100 to ∼40 mm. Figure 1b shows the photo images of the reduced size gas sensor. The left image shows the gas channel hole with diameter ∼1 mm where the IR light will travel through. The right image presents the assembly of the gas sensor. The effective region of the gas channel consists of the 1-mm-diameter gas channel hole and the 40-mm-long gas channel length. For this reduced size gas sensor, a bare die emitter without reflector is used. As the heater area of the thermal emitter is a square shape with dimensions 0.8 mm × 0.8 mm while the gas channel is circular of diameter ∼1 mm, a small portion of radiation from the heater at its 4 corners will be blocked from entering the gas channel. To couple as much radiation emitted from the thermal emitter into the small gas channel, we place the heater surface as close as possible to the gas channel hole, with an optical filter inserted in front of the gas channel hole to allow only radiation of ∼4.26 μm wavelength to enter the gas channel. With the IR rays that enter the gas channel limited to only ∼4.26 μm wavelength, this will help to reduce the unwanted increase in temperature in the gas channel that might occur due to IR ray interaction with the gas molecules in the small channel. The walls of the gas channel are made of Al coated with a layer of gold which are good thermal conductors and could also help to conduct away some of this unwanted change in temperature in the inside of the gas channel. Meanwhile, on the other end of the gas channel, the detector is placed as close as possible to the gas channel hole to collect as much radiation flux as possible coming out of the gas channel. Jigs are designed for placement of the emitter and detector such that the center of the emitter’s heater source and the detector’s sensing is positioned to face the 1-mm-diameter gas channel hole on both ends, held together by micromachined fixtures and screws. There are 2 holes on top of the gas channel for gas inlet and outlet. The CO_2_ gas enters through the gas inlet near the emitter end, travels through the gas channel, and exits through the gas outlet near the detector end via gas tubes of outer diameter 3.175 mm which are inserted into the inlet and outlet holes. This gas channel can be cleaned by purging with N_2_ gas or by isopropyl alcohol (C_3_H_8_O). The total volume of the gas channel is reduced by ∼65 times, from ∼1.96 cm^3^ to ∼0.03 cm^3^.

### CO_2_ Gas Response Measurement

The CO_2_ gas sensor consists of the thermal emitter (Axetris, EMIRS50 AT06V), the optical filter, the gas channel, and the 20% ScAlN-based pyroelectric detector with AOGs. The thermal emitter is set to generate a square wave with peak-to-peak voltage of 2.6 V and duty cycle (50%) at a frequency of 17.4 Hz. The pyroelectric detector is connected to a current amplifier with gain set at 10^9^ V/A, which is connected to a lock-in amplifier to capture the gas response signal. As the lock-in amplifier is set to read only signals modulated at 17.4 Hz frequency, it could help to further reduce an unwanted change in temperature (which does not occur at a specific frequency) caused by interaction between the IR rays and the gas molecules in the small gas channel. The gas channel contains inlet and outlet holes for CO_2_ gas and synthetic air to pass through. The gas measurement is done in a dry laboratory environment with the room temperature at ∼22 °C.

## Results and Discussion

### 20% ScAlN Pyroelectric Films with Abnormally Oriented Grains

**Figure 2:**
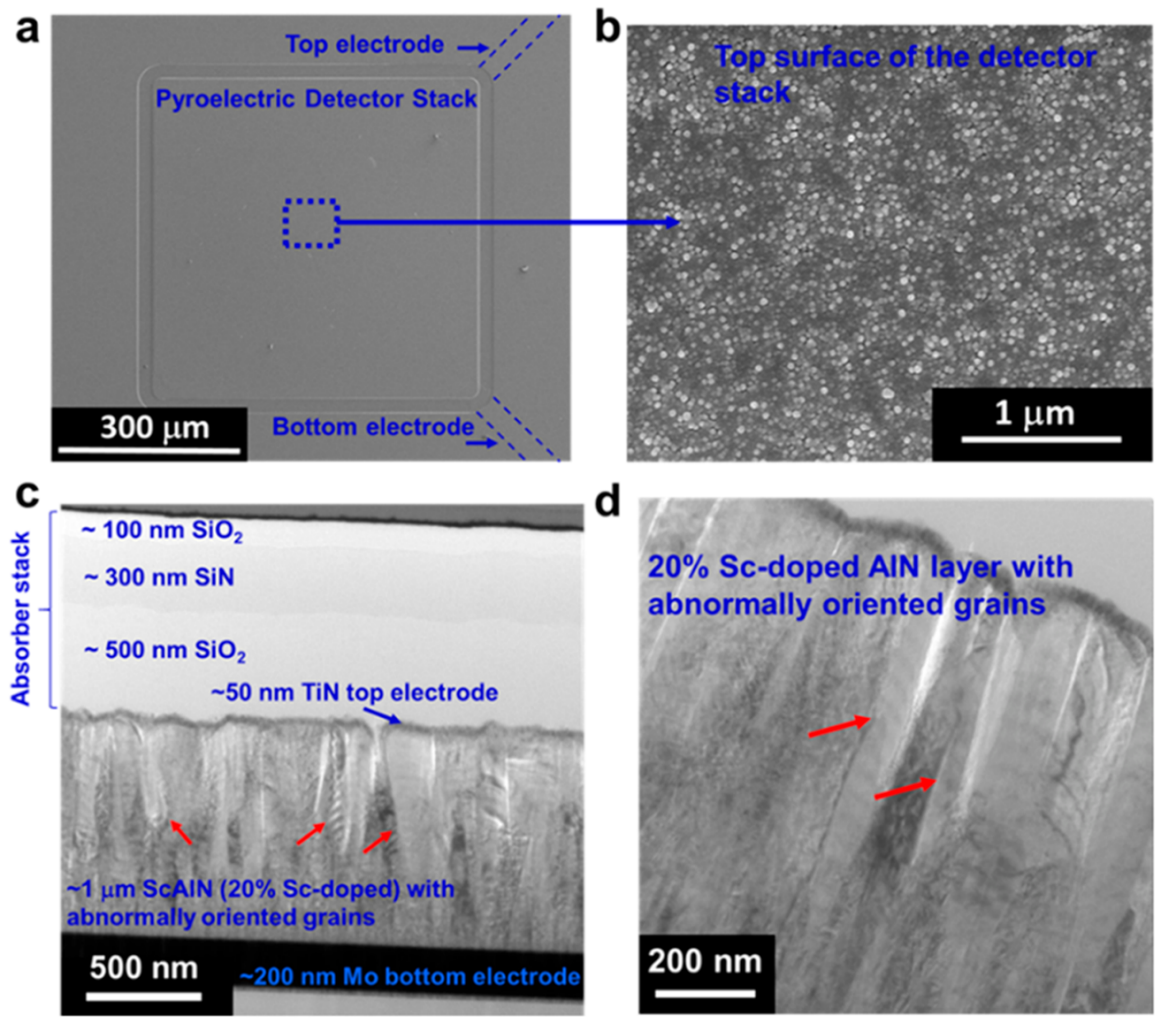
Top view SEM images of (a) the pyroelectric detector device. (b) The top surface shows grainy rough morphology, likely caused by 20% ScAlN with AOGs beneath. Cross sectional TEM images show (c) the different layer stacks in the pyroelectric detector with ∼1-μm-thick ScAlN pyroelectric sensing layer sandwiched between the Mo bottom electrode and TiN top electrode. On top of the TiN top electrode is an absorber stack of SiO_2_–SiN–SiO_2_. Instead of columnar structures aligned to the *c*-axis on the ScAlN layer, AOGs aligned away from the *c*-axis are observed as indicated by the red arrows. (d) Zoom-in into the ScAlN layer show more obvious AOGs within ScAlN layer.

Figure 2 shows the top-view SEM and cross-sectional TEM images of the 20% ScAlN-based pyroelectric detector fabricated in-house for gas sensing. The top-view SEM of the pyroelectric detector stack of area ∼500 μm × 500 μm (Figure 2a) with the top electrode TiN line and bottom electrode Mo line. They will be connected to Al metal contacts, which will subsequently be wire-bonded and electrically connected to extract the electrical signal from the detector. The pyroelectric detector area of ∼500 μm × 500 μm is the sensing area size. This area will sense the incoming IR radiation flux and translate it to electrical signal output from the detector. Figure 2b shows a zoom-in image of the top surface of the detector stack. The SEM image shows a somewhat grainy rough surface with grain diameters of around tens to hundreds nanometers. This grainy rough surface could be accumulated roughness from the subsequent film layers beneath. To examine the layers underneath the top surface, cross-sectional TEM is used to image the detector stack.

Figure 2c shows its cross-sectional TEM image. We can see an ∼1-μm-thick ScAlN layer sandwiched between the bottom Mo electrode (thickness ∼200 nm) and the top TiN electrode (thickness ∼50 nm). On top of the TiN top electrode is the absorber stack that helps to absorb more light that radiates onto the detector. This absorber stack consists of 3 layers of dielectric: ∼500-nm-thick silicon dioxide (SiO_2_), ∼300 nm silicon nitride (SiN), and ∼100-nm-thick SiO_2_. In addition, we also observe AOGs^31−34^ in the 20% ScAlN layer, as indicated by the red arrows. We note that the interface between the ScAlN pyroelectric layer and the TiN top electrode layer is not smooth. This roughness comes from the ScAlN layer, caused by the AOGs that usually form as the film thickness increases. The grains of the AOGs also grow bigger with increasing film thickness, attributed to the grainy surface observed at the top layer of the detector in Figure 2b and the rough interface between the ScAlN pyroelectric layer and the TiN top electrode layer. In cases when there are no AOGs, the ScAlN layers would have presented columnar structures in the *c*-axis orientation. With AOGs, some of the crystal structures are oriented away from the *c*-axis. Figure 2d shows a higher magnification TEM image of the ScAlN layer where we observe the AOG boundaries (indicated by the red arrows) clearly distinguished among the rest of the ScAlN film. A closer look at the top surface of the ScAlN film shows “bumps” of size ∼100–200 nm. These “bumps” from the AOGs subsequently contribute to the rough surface of the detector as seen in Figure 2b.

As the doping concentration of Sc increases in AlN films, the chances of AOG formation increases. In fact, it has been noted that higher Sc concentration usually results in increased density of AOGs in the film.^35^ AOGs usually nucleate midway during the film growth, grow larger as the film thickness increases, and occur in the upper part of the film. They are aligned away from the film’s *c*-axis orientation and usually present themselves as bigger grains, increasing the film’s surface roughness, as noted from Figure 2.

**Figure 3:**
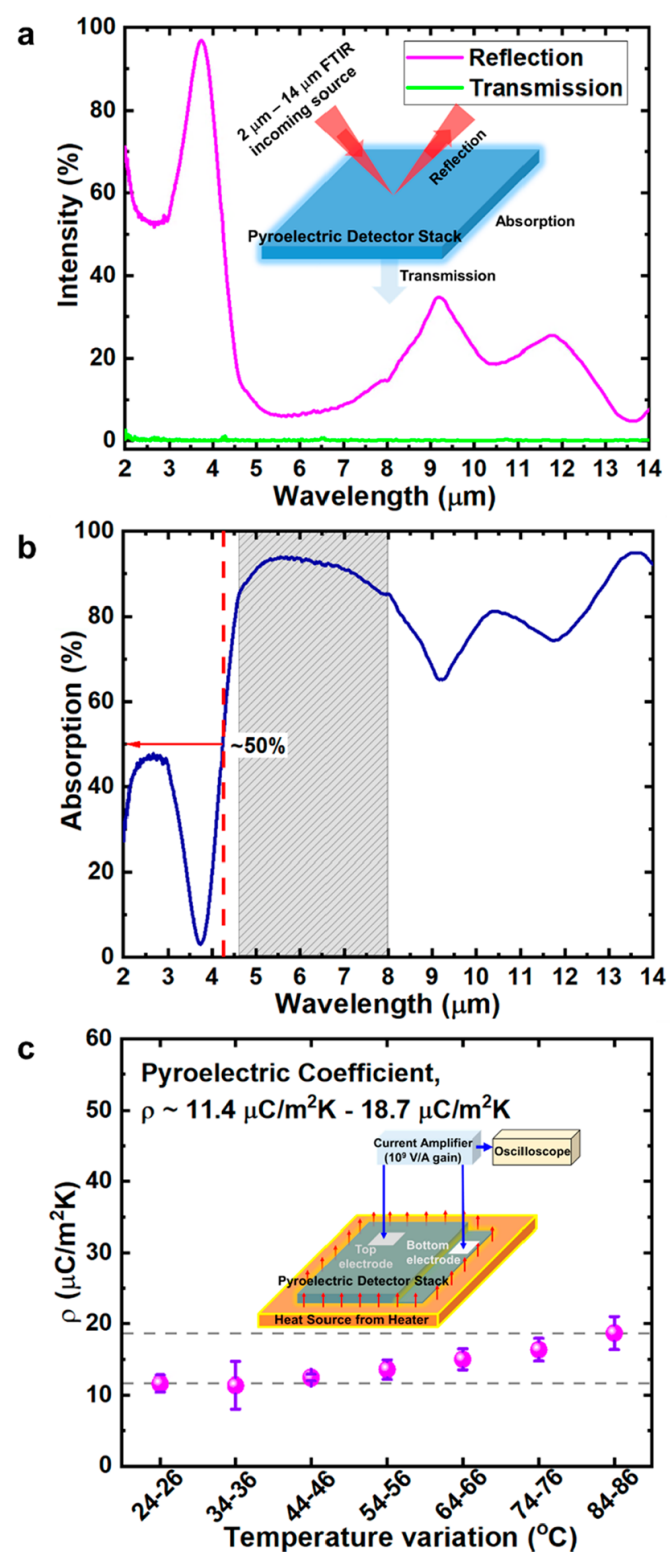
(a) FTIR measurement spectra showing the reflection and transmission spectra of the 20% doped ScAlN pyroelectric detector stack. The transmission spectrum is ∼0% (negligible) across the wavelength range of 2–14 μm due to the 200-nm-thick Mo bottom electrode present in the detector stack. The reflection spectrum shows high reflection at around a 4 μm wavelength. Inset shows a simple schematic of how the FTIR light source impinges on the detector stack that results in transmission, absorption, and reflection. (b) The FTIR absorption spectrum of the 20% doped ScAlN pyroelectric detector stack. The results show around 50% absorption at an ∼4.26 μm wavelength which is the strongest CO_2_ characteristic absorption line with negligible overlap with the absorption peaks from other gases commonly present in ambient air. High absorption (≥85%) is observed at the wavelength range ∼4.6–8 μm. (c) The pyroelectric coefficient, ρ, of 20% ScAlN with AOGs calculated from the measured pyroelectric signal output across the different background temperatures, showing an increasing ρ as the temperature increases with ρ ≈ 11.4–18.7 μC/m^2^ K. Inset shows a schematic on how the pyroelectric signal is extracted.

Figure 3 shows the optical and electrical characteristics of the 20% ScAlN pyroelectric detector stack with AOGs. Figure 3a shows the Fourier Transform Infrared (FTIR) measured reflection and transmission spectra of this detector stack across wavelengths range 2–14 μm. The inset shows that when light impinges on the pyroelectric detector stack, it will either transmit through the stack, reflect from the stack, or be absorbed by the stack. In Figure 3a, the transmission spectrum measured shows negligible (∼0%) intensity which is probably caused by the ∼200-nm-thick Mo bottom electrode layer toward the bottom of the detector stack that blocks the light from transmitting. On the other hand, the reflection spectrum shows high reflection in the ∼4 μm wavelength region. This will subsequently affect the absorption intensity at the same wavelength region, as absorption is calculated by subtracting the measured FTIR transmission and reflection spectra across wavelengths in the range 2–14 μm from 100%.^3,30,36^

Figure 3b shows an FTIR absorption spectrum of this detector stack. The drop in absorption at around the 4 μm wavelength region is mostly due to the high reflection of the detector stack in the same wavelength region as depicted in Figure 3a. For our CO_2_ gas sensing application, we hope that absorption will be as high as possible for the detector to receive as much optical signal change due to slightest change in gas concentration, which will subsequently increase the signal-to-noise ratio translated to electrical signal output from the detector. In Figure 3b, the absorption is ∼50% at ∼4.26 μm wavelength. This is our wavelength region of interest where the CO_2_ characteristic absorption line occurs. The absorption of ∼50% for the 20% ScAlN-based pyroelectric detector with AOGs is lower than that of the 12% ScAlN-based pyroelectric detector previously reported with absorption ∼75%^3,29,36^ but higher than that of AlN-based pyroelectric detector which reports absorption ∼25%.^30,36^ The high reflection at the ∼4 μm wavelength region which causes lower absorption in the same wavelength region could be caused by the rougher surface^37^ of the detector stack (as observed from Figure 2b previously) mainly attributed by the AOGs in the 20% ScAlN layer that induces the rough interface between the ScAlN layer and TiN (as observed from Figure 2c and d previously).

Meanwhile, we observe absorption ≥85% from the wavelength range ∼4.6 to 8 μm. This is useful information when considering detecting gases with characteristic absorption lines falling in this wavelength range such as nitric oxide (NO), nitrogen dioxide (NO_2_), methane (CH_4_), and hydrogen sulfide (H_2_S).^38^ However, it is also worthwhile to take note that while we consider the detector’s absorption across the mid-IR wavelength range, we should also take note of the gas absorbance in this wavelength range, as different gases have difference absorbance. According to the National Institute of Standards and Technology (NIST) Chemistry WebBook (NIST Standard Reference Database Number 69), CO_2_ has an absorbance of ∼1.8^39^ at the ∼4.26 μm wavelength, while NO has absorbance of ∼0.63^40^ at the ∼5.4 μm wavelength, CH_4_ has absorbance of ∼0.36^41^ at the ∼7.7 μm wavelength, and H_2_S has absorbance of ∼0.18^42^ at the ∼7.5 μm wavelength. Out of these 4 gases, CO_2_ has the highest absorbance, indicating that it absorbs more IR radiation at the ∼4.26 μm wavelength compared to NO, CH_4_, and H_2_S at their characteristic absorption wavelengths, respectively. The absorbance of CO_2_ is ∼3× that of NO and ∼10× that of H_2_S. Hence, even though the detector’s absorption at the ∼4.26 μm wavelength is ∼50% (Figure 3b), the high absorbance of CO_2_ gas allows it to create enough of a drop in the radiation flux to be picked up by the pyroelectric detector.

In addition to the detector’s absorption and gas absorbance, the pyroelectric coefficient of the detector is also a factor that determines the output signal received. In this work, we are using >20% Sc-doped AlN compared to the previously reported^3^ 12% Sc-doped AlN pyroelectric material. Our previous work^27^ has shown that the specific detectivity of the detector increases when doped with 12% Sc, and we anticipate a further increase in specific detectivity as the Sc doping increases. Meanwhile, Akiyama et al.^43^ was examining the piezoelectric response of ScAlN and noted an increase in the piezoelectric response as the Sc doping concentration increases up to 43% Sc concentration. The piezoelectric response starts to drop when the Sc concentration increases beyond 43%. A similar trend for the pyroelectric response in Sc-doped AlN might also occur.

We further estimate the pyroelectric coefficient (ρ) of this 20% ScAlN with AOGs based on the following equation:^27^
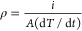
1where *i* is the current measured, *A* is the sensing area of the 20% ScAlN pyroelectric detector stack with AOGs which is ∼0.29 mm^2^, d*T* is the temperature change, and d*t* is the rise time of the detector when encountering the temperature change. Five readings of current output were taken at each temperature variation of ∼24–26 °C to ∼84–86 °C. Figure 3c shows a plot of ρ calculated for the 2 °C temperature change across different temperature settings. The inset shows the schematic of how the pyroelectric output signal is extracted. The pyroelectric coefficient of 20% ScAlN pyroelectric detector with AOGs calculated ranges from ∼11.4 to ∼18.7 μC/m^2^ K, higher than what was previously reported for the 12% ScAlN pyroelectric detector.^27,44^ This is, in general, in line with the expectation that higher Sc doping concentration gives higher pyroelectric coefficient, since it has previously been demonstrated^43^ that the higher Sc doping concentration resulted in an increase in the piezoelectric response of ScAlN.

The pyroelectric coefficients of AlN and Sc-doped AlN films reported so far with different Sc doping concentrations compared with the pyroelectric coefficient of our current 20% ScAlN are shown in Table S1. We note a general increase in the pyroelectric coefficient as the Sc doping concentration increases up to 35%. For Sc doping concentration above 35%, to the best of our knowledge, there has been no report so far. When AlN is doped with Sc, Sc will replace Al to form ScAlN. With more Sc substituting Al to form ScAlN, the increased Sc–N bonds cause the original AlN wurzite crystal lattice to soften its elastic constant and increase in piezoelectric stress coefficient, leading to an increase in piezoelectric strain. Structurally, a decrease in the *c*/*a* ratio of wurtzite structure toward the *c*/*a* ratio of hexagonal boron-nitride has been observed^45^ as the Sc concentration increases.

If we consider the following equation^44,46^ on effective pyroelectric coefficient (ρ^eff^)
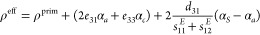
2where ρ^prim^ = primary pyroelectric effect, *e*_*ij*_ = piezoelectric stress coefficients in Voigt notation, α_*a*_ and α_*c*_ = anisotropic linear thermal expansion, *d*_31_ = transversal piezoelectric strain coefficient, *s*_11_^*E*^ and *s*_12_^*E*^ = elastic compliance coefficient of ScAlN, and α_*S*_ = linear thermal expansion of substrate; the increase in Sc substitution of Al will result in softening of elastic constants leading to a reduction in *s*_11_^*E*^ and *s*_12_^*E*^, increase in piezoelectric stress coefficients (*e*_31_ and *e*_33_), and piezoelectric strain (*d*_31_). The terms (2*e*_31_α_*a*_ + *e*_33_α_*c*_) and  will then increase, leading to increase in effective pyroelectric coefficient.

Although in general pyroelectric coefficients of ScAlN increases with increasing Sc doping concentration, this value might vary across films prepared by different deposition methods due to film and substrate properties, and impurity levels in the films.^44^ The pyroelectric coefficient of our 20% ScAlN pyroelectric detectors show ∼11.4 μC/m^2^ K at room temperature, slightly higher than those reported in the literature of ∼20% Sc-doped AlN.^44,47^ Kurz et al.^44^ reported a pyroelectric coefficient of ∼8 μC/m^2^ K at room temperature for 22% Sc-doped AlN and Bette et al.^47^ reported pyroelectric coefficient ∼9.7 μC/m^2^ K for 27% Sc-doped AlN.

We believe that our higher pyroelectric coefficient for 20% Sc-doped AlN at room temperature could be due to the larger grains in the films caused by the AOGs, as it has been previously demonstrated^48^ that pyroelectric coefficient changes with variation in average grain size, with increasing grain size exhibiting an increasing pyroelectric coefficient.^49^ In addition to the higher pyroelectric coefficient of our 20% ScAlN pyroelectric detectors measured at room temperature, we also note that its pyroelectric coefficient seems to have temperature dependence characteristics, showing an increasing trend across different background temperatures. This is most likely also caused by the presence of AOGs in the 20% ScAlN film. The larger grains from the AOGs result in the pyroelectric coefficients being temperature dependence.^48^ On top of that, factors such as impurities in the films^50^ and stress and strain^51^ induced in the films could also contribute to the temperature dependence characteristics of pyroelectric coefficients. The observation of a higher pyroelectric coefficient for 20% Sc-doped AlN with AOGs at room temperature and its temperature dependence pyroelectric coefficient characteristics in this work will provide a reference to help us understand more on the pyroelectric characteristics of ScAlN films with AOGs which remains relatively unexplored.

### NDIR CO_2_ Gas Response

**Figure 4:**
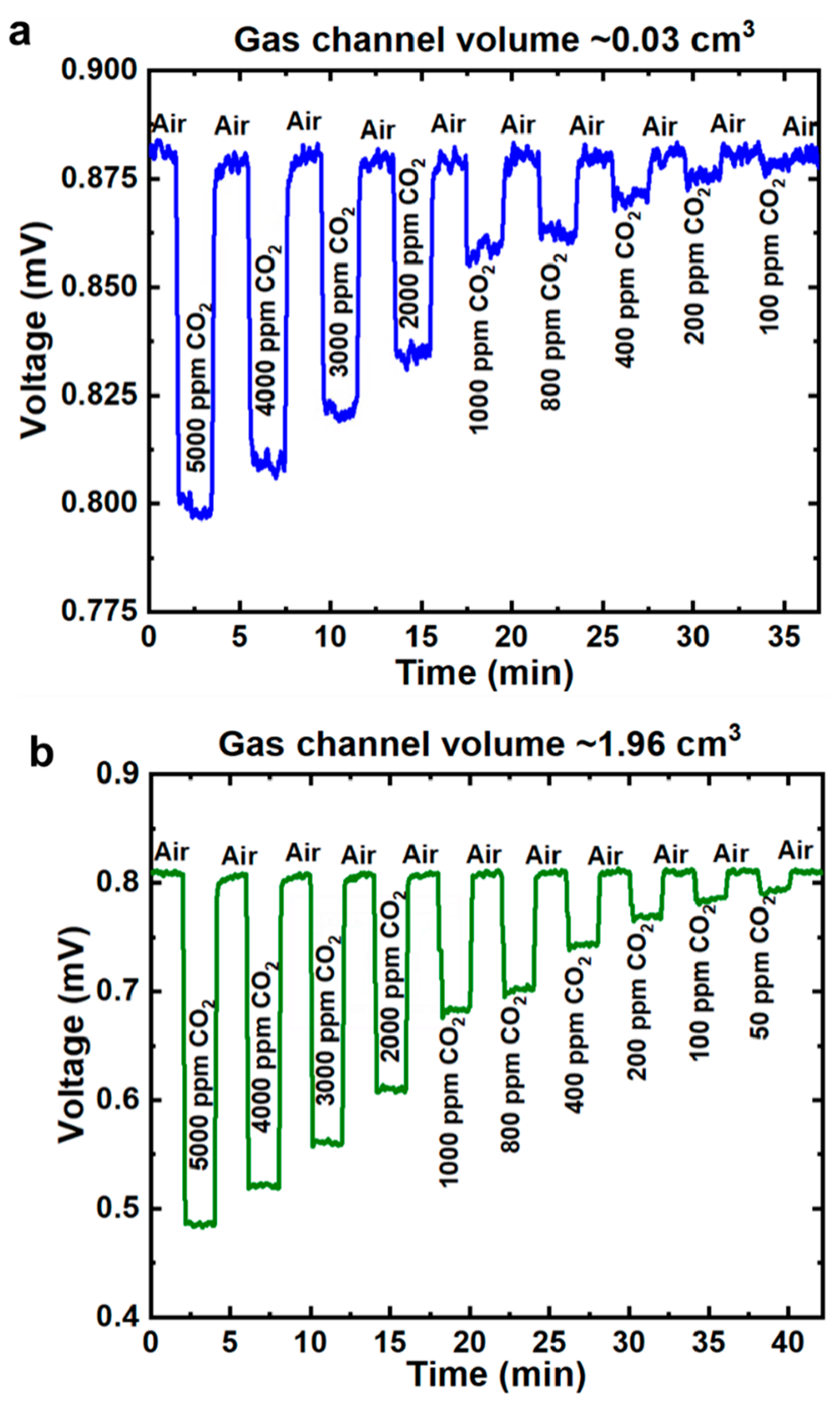
Voltage readings due to different CO_2_ gas concentrations between synthetic air and CO_2_ gas cycled at 2 min intervals for (a) gas channel 40 mm length, 1 mm diameter, ∼0.03 cm^3^ volume from 5000 ppm of CO_2_ gas concentration down to 100 ppm of CO_2_ gas concentration; and (b) gas channel 100 mm length, 5 mm diameter, ∼ 1.96 cm^3^ volume from 5000 ppm of CO_2_ gas concentration down to 50 ppm of CO_2_ gas concentration.

Figure 4 shows the gas responses of the gas sensors to CO_2_ gas at different gas concentrations. The gases are cycled between synthetic air and CO_2_ gas at 2 min intervals. Figure 4a shows the CO_2_ gas responses for the miniaturized gas channel of volume ∼0.03 cm^3^, while Figure 4b shows that for the larger gas channel of volume ∼1.96 cm^3^. For the smaller gas channel, the gas sensor shows responses from 5000 ppm down to 100 ppm (Figure 4a), with CO_2_ gas response ranging from ∼0.3% to ∼9.4%. This is in general sufficient for practical air quality monitoring. For the bigger gas channel, the gas sensor shows responses from 5000 ppm down to 50 ppm (Figure 4b), with CO_2_ gas response ranging from ∼2.3% to ∼40%. Though the data here only show measurements down to 50 ppm for the bigger gas channel sensor, it should be able to sense lower than 50 ppm of CO_2_ concentration. The global average atmospheric CO_2_ levels have been seeing an increasing trend, from ∼390 ppm in Year 2010 to 412.5 ppm^52^ reported in Year 2020. Indoors, when the CO_2_ gas concentration increases to ∼1000 ppm and higher, SBS also kicks in where occupants will experience respiratory issues, dizziness, drowsiness, and headaches with degraded performances in problem solving and decision making.^53^

**Figure 5:**
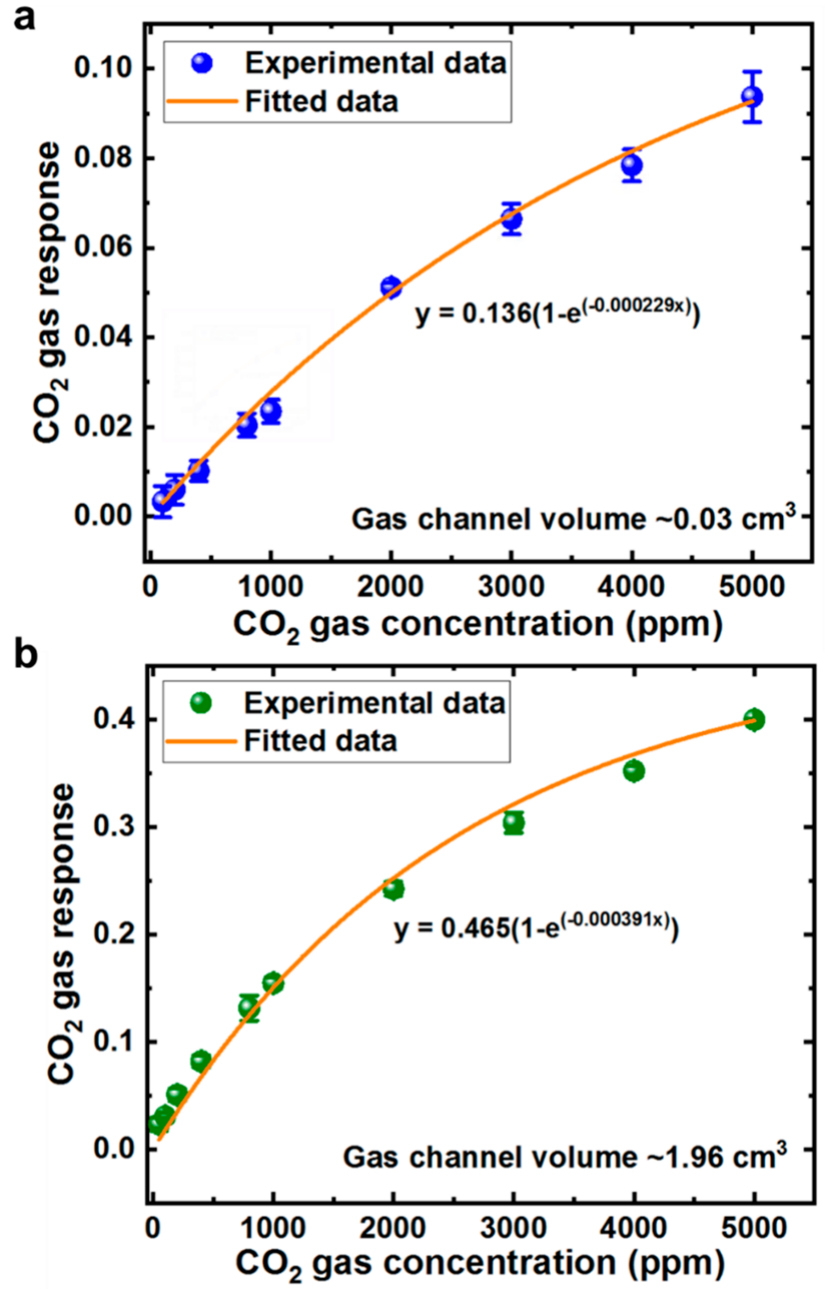
CO_2_ gas response for (a) gas channel volume ∼0.03 cm^3^ and (b) gas channel volume ∼1.96 cm^3^ across different CO_2_ gas concentrations up to 5000 ppm. Modified Beer–Lambert equation is used to fit the experimental data.

We calculate the ratio of CO_2_ gas response (voltage change due to CO_2_ shown in Figure 4) to synthetic air and plot it against respective CO_2_ gas concentrations as shown in Figure 5. Figure 5a shows the experimental CO_2_ gas response plotted against different CO_2_ gas concentrations for the small ∼0.03 cm^3^ volume gas channel, while Figure 5b shows that for the bigger ∼1.96 cm^3^ volume gas channel. We note that across different CO_2_ gas concentrations, the CO_2_ gas response measured for the smaller gas channel is lower than that of the bigger gas channel. Five readings of CO_2_ gas response are taken, and the average and data range are calculated at each gas concentration.

We try to fit the experimental data using the modified Beer–Lambert’s equation:^5,54^
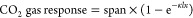
3where *x* is the CO_2_ gas concentration, κ is the effective absorption coefficient of CO_2_, *l* is the optical path length in the gas system, which is ∼0.04 m for the small gas channel and ∼0.1 m for the bigger gas channel, and “span” is a coefficient which is an indication on the amount of IR radiation that can be absorbed. The experimental data fitted well with the equation, with coefficient of determination (*R*^2^) of both gas channels >0.99 (∼0.9925 for the small gas channel and ∼0.9976 for the big gas channel).

With the fitted data, we try to compare the various parameters between the ∼0.03 cm^3^ volume and the ∼1.96 cm^3^ volume gas channels. The comparison of the fitted data using eq 3 for both gas channels with ∼0.03 cm^3^ volume and ∼1.96 cm^3^ volume is shown in Table S2. The optical path lengths, *l*, used are based on the gas channel lengths with 0.04 m for the smaller gas sensor and 0.1 m for the bigger gas sensor. “Span” differs between gas channels, and due to the smaller volume of the ∼0.03 cm^3^ gas sensor, less IR radiation is able to pass through it, hence less CO_2_ absorption, resulting in lower span compared to the ∼1.96 cm^3^ gas sensor. The amount of IR radiation that can be absorbed in the small gas sensor is less than the big gas sensor which has greater volume, and hence will saturate faster than the bigger gas sensor. Nevertheless, the effective absorption coefficient of the smaller gas sensor is higher (∼0.00573 m^–1^) compared to that of the bigger gas sensor (∼0.00391 m^–1^), indicating more CO_2_ absorption per unit m in the smaller gas sensor.

**Figure 6:**
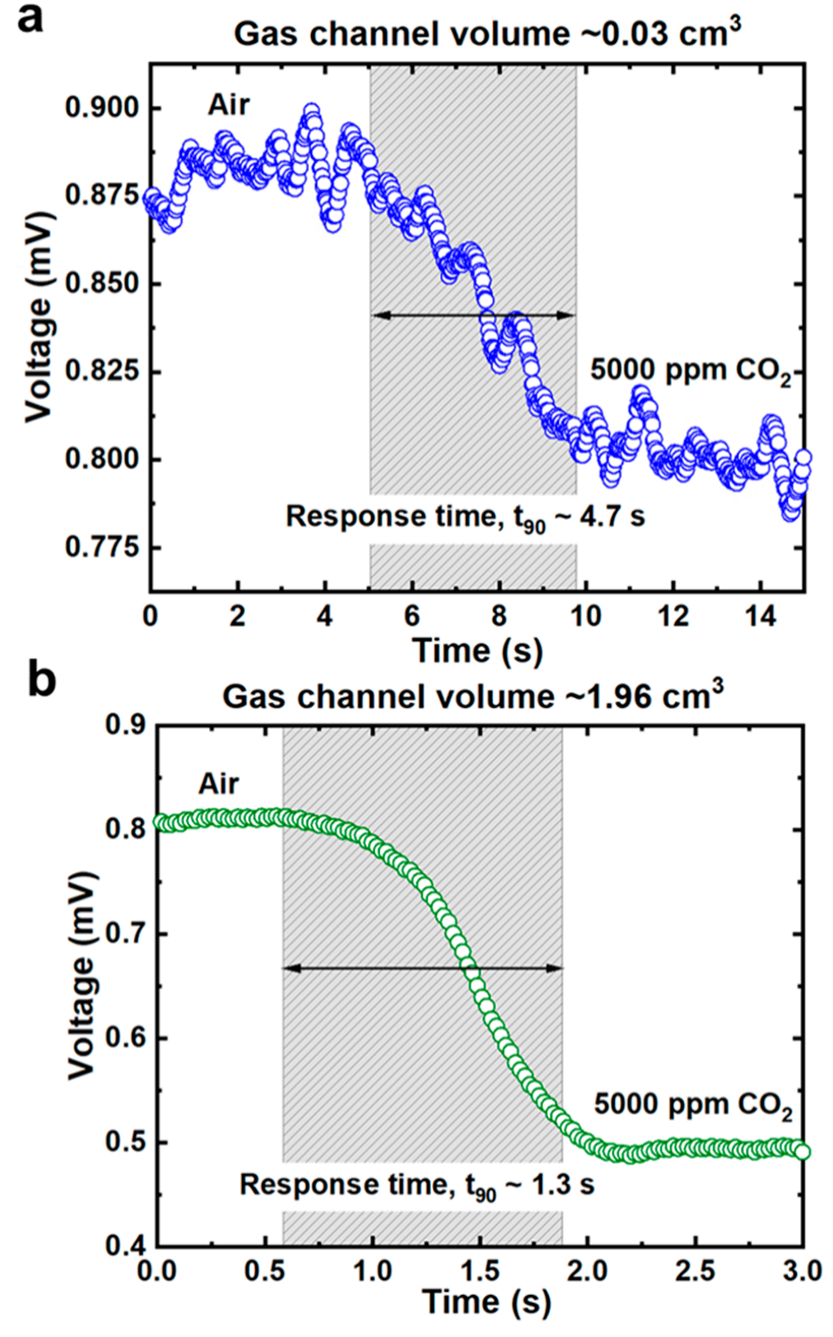
Response times, *t*_90_, taken for gas sensor to detect CO_2_ gas from the initial state of synthetic air. Measurement is taken at 5000 ppm of CO_2_ gas concentration for (a) ∼0.03 cm^3^ gas channel volume which gives *t*_90_ ∼ 4.7 s and (b) ∼1.96 cm^3^ gas channel volume which gives *t*_90_*∼* 1.3 s.

Figure 6 shows the time taken for the gas sensors to detect 5000 ppm of CO_2_. We take the response time as *t*_90_, which is the time taken for the gas sensor to detect a 90% change in the gas concentration. The response time, *t*_90_, is ∼4.7 s for the smaller gas sensor (∼0.03 cm^3^ volume) and ∼1.3 s for the bigger gas sensor (∼1.96 cm^3^ volume). Although it takes around ∼3 s slower for the smaller gas sensor to detect the change in the gas concentration, the overall response times for both gas channels are around 5 s or less. This response time is considered fast for a gas sensor working at room temperature ∼22 °C environment, as most gas sensors operating at room temperature have longer response time.^6,9,55^

With the smaller gas sensor which is 65× smaller in volume compared to the bigger gas sensor, we manage to obtain the lowest detection limit of 100 ppm of CO_2_ gas concentration and *t*_90_ ∼ 4.7 s, suitable for environmental monitoring. To the best of our knowledge, this is the first demonstration of a functional small NDIR CO_2_ gas sensor using a 20% ScAlN-based pyroelectric detector. Table 1 shows a comparison of the size and performance of recently reported NDIR CO_2_ gas sensors using broadband sources and microelectromechanical systems (MEMS) detectors for environmental monitoring, published within the past 2 years.^3,5,6,56−58^ For NDIR gas sensors which are known for their large footprints, size reduction could bring about a compromise in the lowest detection limit and response time. This can be seen in Table 1 by an NDIR gas sensor^5^ with lowest CO_2_ detection limit reported at 2 ppm. This sensor is however of larger footprint with length 448 mm and diameter ∼72 mm. Compared with other recently reported NDIR CO_2_ gas sensors using broadband sources and MEMS detectors, and with its size taken into account, the detection limit and response time of our smaller gas sensor are comparable, if not better.

**Table 1:** Summary of Size and Performance of Recently Reported NDIR CO_2_ Gas Sensors Using Broadband Sources and MEMS Detectors for Environmental Monitoring, Including Our Current Work

Source	Detector	Lowest measured CO_2_ concentration (ppm)	Response time (s)	Gas channel diameter (mm)	Distance between source and detector (mm)	Year of Reference
Filament Lamp	Thermopile	400	60	26	30–40	2021,^6^ 2020^56^
MEMS IR emitter	Thermopile	0.5%	10	∼3	∼10	2021^57^
IR Light	LiTaO_3_ Pyroelectric	2	Information not available	∼72	448	2020^5^
Broadband light	LiTaO_3_ Pyroelectric	Measured Range: 0–20%	<11	8	108	2022^58^
Thermal Emitter	12% ScAlN Pyroelectric	25	<2	5	100	2021^3^
Thermal Emitter	20% ScAlN Pyroelectric (with AOGs)	100	∼5	1	40	Current work

We also check the smaller gas sensor’s selectivity to gases other than CO_2_. In our testing measurement, CO_2_ is mixed with synthetic air. Here, we replace synthetic air with N_2_ and 49% SF_6_, respectively, to determine the gas sensor’s response when CO_2_ is mixed with other gases.

**Figure 7:**
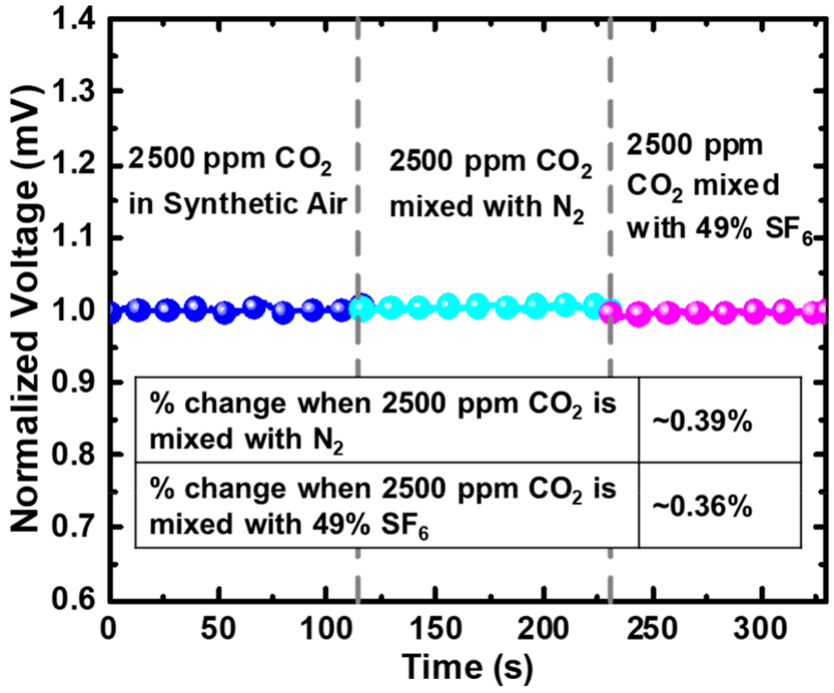
Normalized voltage measured using the smaller gas sensor (∼0.03 cm^3^ channel volume) when 2500 ppm of CO_2_ gas is in synthetic air (blue plot), mixed with N_2_ gas (cyan plot) and mixed with 49% SF_6_ gas (magenta plot), respectively. The voltage readings are normalized to that of 2500 ppm of CO_2_ in synthetic air. Inset shows the percentage change in the voltage when 2500 ppm of CO_2_ is mixed with N_2_ and 49% SF_6_, respectively, compared to 2500 ppm of CO_2_ in synthetic air.

Figure 7 shows the voltage detected when CO_2_ gas concentration of 2500 ppm is mixed with synthetic air, N_2_, and 49% SF_6_ gas, respectively. The vertical axis is normalized to 2500 ppm of CO_2_ gas in synthetic air. We note that the selectivity of CO_2_ to N_2_ and SF_6_ seems high, with the voltage readings approximately the same for 2500 ppm of CO_2_ in synthetic air, 2500 ppm of CO_2_ in N_2_, and 2500 ppm of CO_2_ in 49% SF_6_. Nevertheless, there seems to be some slight deviation in voltage readings when CO_2_ is in N_2_ and in SF_6_ compared to CO_2_ in synthetic air.

We further calculate the percentage change in voltage when CO_2_ is in N_2_ and CO_2_ is in SF_6_ compared to CO_2_ in synthetic air. As shown by the inset in Figure 7, we calculate a voltage change of ∼0.39% when CO_2_ is mixed with N_2_ and a voltage change of ∼0.36% when CO_2_ is mixed with 49% SF_6_. The percentage of voltage changes influenced by N_2_ and 49% SF_6_ is minimum or negligible. This is most probably due to the optical filter that allows light of ∼4.26 μm wavelength to pass through, which is the wavelength at which CO_2_ gas absorbs, with mostly CO_2_ absorption peaks and negligible absorption peaks from other gases.^59^

Finally, we did a comparison of our miniaturized CO_2_ gas sensor with commercial off-the-shelf CO_2_ gas sensors targeted for environmental monitoring applications. Table 2 shows 6 off-the-shelf CO_2_ gas sensors based on different detection principles in addition to our miniaturized CO_2_ gas sensor. The off-the-shelf CO_2_ gas sensors are based on photoacoustic spectroscopy (PAS), metal oxide, and NDIR principles. The upper limit of their measurement ranges can go until 2000 ppm or as high as 65000 ppm, while the lower limit of their measurement ranges start from 0 or 400 ppm. As CO_2_ levels in ambient are at around 400 ppm, some CO_2_ gas sensors range start at 400 ppm. Response time of the sensors are typically tens of seconds with 1 metal oxide CO_2_ gas sensor having a response time of ∼1 s. As for interference due to other gases, this information is not available for most off-the-shelf CO_2_ gas sensors. We note that metal oxide CO_2_ gas sensors did mention interference from other gases which might cause poisoning to the sensor sensing layer or that baselining is required to mitigate the interference from other gases. Comparing our NDIR miniaturized CO_2_ gas sensor with these off-the-shelf CO_2_ gas sensors, we find our measurement range to be 100–5000 ppm. The lower detection limit is less than 400 ppm, which is the CO_2_ concentration in the environment. We would also like to highlight that our miniaturized CO_2_ gas sensor is capable of sensing beyond 5000 ppm, but in this paper, measurement was done up until 5000 ppm. For response time (*t*_90_), our miniaturized CO_2_ gas sensor is ∼5 s, which is lower than most of the off-the-shelf CO_2_ gas sensors presented in Table 2. As for interference caused by other gases, although this information is not readily available for most of the off-the-shelf CO_2_ gas sensors, our miniaturized CO_2_ gas sensor has shown (in Figure 7) negligible interference caused by N_2_ and SF_6_.

**Table 2:** Comparison of Our Miniaturized CO_2_ Gas Sensor with Commercial off-the-Shelf CO_2_ Gas Sensors

Sensor Brand	Principle	Range (ppm)	Response Time (s)	Interference from other gases
Infineon (XENSIV PAS CO2^60^)	PAS	0–32000	*t*_63_ ∼ 90	Information not available
Sensirion (SCD42^61^)	PAS	0–40000	*t*_63_ ∼ 60	Information not available
SGX Sensortech (MiCS-VZ-89TE^62^)	Metal Oxide	400–2000	Equivalent to conventional NDIR CO_2_ sensors	Yes (some gases will cause poisoning)
ScioSense (ENS160^63^)	Metal Oxide	400–65000	*t*_63_ ∼ 1	Yes (requires baselining)
Alphasense (IRC-A1^64^)	NDIR	0–5000	*t*_90_ < 40	Information not available
GSS (CozIR-A^65^)	NDIR	0–2000, 0–5000, 0–10000	*t*_90_ ∼ 30	Information not available
Current work	NDIR	100–5000	*t*_90_ ∼ 5	Negligible to N_2_, SF_6_ (Refer to Figure 7)

## Conclusions

We have successfully downsized an NDIR CO_2_ gas sensor ∼65 times from ∼1.96 cm^3^ volume down to ∼0.03 cm^3^ volume. A pyroelectric detector which contains 20% ScAlN with AOGs is used as the detecting device. The pyroelectric detector containing the 20% ScAlN sensing layer with AOGs is characterized optically and electrically. The absorption of the pyroelectric detector stack shows ∼50% at 4.26 μm wavelength which is the CO_2_ characteristic absorption wavelength. For 20% Sc-doped AlN with AOGs, the pyroelectric coefficient calculated from the measured output electrical signal shows a higher pyroelectric coefficient of ∼11.4 μC/m^2^ K. While deriving the pyroelectric coefficient of 20% ScAlN with AOGs, we observe an increasing trend in pyroelectric coefficient, as the background temperature increases while we keep the temperature fluctuation at 2 °C. This could most probably be due to AOGs in the ScAlN layer. The larger grains from the AOGs could be one of the factors contributing to the temperature dependence characteristics of 20% ScAlN in this work. The gas responses of both the big and small gas sensors are measured at different levels of CO_2_ gas concentrations. Both are able to reach gas concentrations down to 100 ppm, a concentration suitable for practical monitoring of CO_2_ levels in the environment. The experimental CO_2_ gas response data are fitted using modified Beer–Lambert’s equation, and a comparison is done between both gas sensors. We note that although the smaller gas sensor has smaller volume, which restricts the amount of IR radiation that can travel in the gas channel, reducing the amount of CO_2_ gas to be absorbed, the effective absorption coefficient of the small gas sensor is higher compared to the big gas sensor. The gas response times measured show that the smaller gas sensor has a response time ∼4.7 s, which is ∼3 s behind that of the bigger gas sensor which measures a response time of ∼1.3 s. Nevertheless, overall response time for both gas sensors fall in the range of ∼5 s or less, which is considered fast in an operational environment of ∼22 °C, as most gas sensors have a response time ranging from several minutes to hours. A comparison is done between this smaller gas sensor and recently published NDIR CO_2_ gas sensors using MEMS emitters and detectors. With the smaller footprint of this gas sensor, its lower detection limit and response time are comparable, if not better than the rest being compared. This smaller gas sensor is further tested in CO_2_ mixed with N_2_ and SF_6_, respectively, to check the selectivity of this gas sensor to CO_2_. We noted high selectivity with CO_2_ based on using N_2_ and SF_6_ with a voltage variation at ∼0.4% compared to CO_2_ mixed in synthetic air. In addition to exploring the pyroelectric characteristics of 20% doped ScAlN with AOGs, this paper also demonstrates an NDIR CO_2_ gas sensor device downsized in volume by ∼65 times, targeting the miniature CO_2_ gas sensor that could one day be integrated onto mobile phones or consumer products for convenient real-time air quality monitoring, which is especially of importance in these times of COVID-19.

## Supporting Information Available

Summary of pyroelectric coefficients with different Sc doping concentrations reported so far in the literature (Table S1), Comparison of fitted data based on CO_2_ gas response experimental data at different CO_2_ gas concentration using modified Beer–Lambert’s equation for both smaller and larger gas channel (Table S2) (PDF)
