# Supplementary material for: Miniaturized CO2 Gas Sensor Using 20% ScAlN-Based Pyroelectric Detector
Source: ACS Sens. 2022 Aug 9;7(8):2345–57. doi: 10.1021/acssensors.2c00980 (PMC9425554; doi:10.1021/acssensors.2c00980)
Supplement: Supplementary file 1 — se2c00980_si_001.pdf [file se2c00980_si_001.pdf]

Supporting Information

Miniaturized CO_2 Gas Sensor using 20% ScAlN-based Pyroelectric Detector

Doris Keh Ting Ng,[*,a] Linfang Xu,[a] Weiguo Chen,[a] Huanhuan Wang,[a] Zhonghua Gu,[a] Xavier Xujie Chia,[a,b] Yuan Hsing Fu,[a] Norhanani Jaafar,[a] Chong Pei Ho,[a] Tantan Zhang,[a] Qingxin Zhang,[a] Lennon Yao Ting Lee[a]

*Email: Doris_NG@ime.a-star.edu.sg

[a]Institute of Microelectronics, A*STAR (Agency for Science, Technology and Research), 2 Fusionopolis Way, #08-02, Innovis Tower, Singapore 138634, Singapore

[b]Photonics Devices and Systems Group, Engineering Product Development, Singapore University of Technology and Design, 8 Somapah Road, Singapore 487372, Singapore

KEYWORDS: pyroelectric detector, scandium aluminum nitride (ScAlN), abnormally oriented grains, CO_2 gas sensor, MEMS, CMOS compatible, non-dispersive infrared

Table S1. Pyroelectric coefficients of AlN and Sc-doped AlN films reported so far with different Sc doping concentrations.

Sc doping concentration (%)	Pyroelectric Coefficient ($\mu C/m^2K$) reported
0 (AlN)	~5.5[1], 6-8[2]
6	~6.2[1]
12	~8.35[3]
14	~8[1]
20	~11.8 (Current work – ScAlN with AOGs)
22	~8.2[1]
27	9.7[4]
30	~9.8[1]
35	19.3[4]

Table S2. Comparison of fitted data based on CO_2 gas response experimental data at different CO_2 gas concentration using modified Beer Lambert's equation for both smaller gas channel and larger gas channel.

Description	CO_2 in synthetic air	
Gas channel volume	$\sim 0.03\ cm^3$	$\sim 1.96\ cm^3$
Fitted equation	$y = 0.136(1 - e^{(-0.000229x)})$	$y = 0.465(1 - e^{(-0.000391x)})$
span	0.136	0.465
κ (*effective absorption coefficient*)	$0.00573\ m^{-1}$	$0.00391\ m^{-1}$
l (*optical path length*)	$0.04\ m$	$0.1\ m$

REFERENCES

[1] Kurz, N.; Lu, Y.; Kirste, L.; Reusch, M.; Žukauskaitė, A.; Lebedev, V.; Ambacher, O. Temperature dependence of the pyroelectric coefficient of AlScN thin films. *Physica Status Solidi a* **2018**, *215*, 1700831.

[2] Fuflyigin, V.; Salley, E.; Osinsky, A.; Norris, P. Pyroelectric properties of AlN, *Applied Physics Letters* **2000**, *77* (19), 3075-3077.

[3] Ng, D. K. T.; Zhang; T.; Siow, L. Y.; Xu, L.; Ho, C. P.; Cai, H.; Lee, L. Y. T.; Zhang, Q.; Singh, N. A functional CMOS compatible MEMS pyroelectric detector using 12%-doped scandium aluminum nitride. *Applied Physics Letters* **2020**, *117*, 183506.

[4] Bette, S.; Fichtner, S.; Bröker, S.; Nielen, L.; Schmitz-Kempen, T.; Wagner, B.; Buggenhout, C. V.; Tiedke, S.; Tappertzhofen, S. Infrared-laser based characterization of the pyroelectricity in AlScN thin-films. *Thin Solid Films* **2019**, *692*, 137623.
